# A web-based video messaging intervention for suicide prevention in men: study protocol for a five-armed randomised controlled trial

**DOI:** 10.1186/s13063-024-08308-1

**Published:** 2024-07-09

**Authors:** Jin Han, Aimy Slade, Hiroko Fujimoto, Wu Yi Zheng, Artur Shvetcov, Leonard Hoon, Joost Funke Kupper, Manisha Senadeera, Sunil Gupta, Svetha Venkatesh, Kon Mouzakis, Yuanyuan Gu, Anam Bilgrami, Noura Saba, Henry Cutler, Philip Batterham, Katherine Boydell, Fiona Shand, Alexis Whitton, Helen Christensen

**Affiliations:** 1 Black Dog Institute, University of New South Wales, Sydney, NSW Australia; 2 Applied Artificial Intelligence Institute, Deakin University, Melbourne, VIC Australia; 3 Centre for the Health Economy, Macquarie University, Sydney, NSW Australia; 4 Centre for Mental Health Research, Australia National University, Canberra, ACT Australia; 5 Centre for Global Health Equity, New York University Shanghai, Shanghai, China

**Keywords:** Suicide, Support services, Men, Video messaging, Service preferences

## Abstract

**Background:**

More than 50% of people who die by suicide have not been in contact with formal mental health services. The rate of people who fly ‘under the radar’ of mental health services is higher among men than women, indicating a need to improve engagement strategies targeted towards men who experience suicidal thoughts and/or behaviours. In Australia, a range of mental health support services exist, designed specifically for men, yet, a substantial proportion of men do not use these services. The aim of this study is to evaluate whether a brief online video-based messaging intervention is an effective approach for encouraging men with suicidal thoughts and/or behaviours to engage with existing support services.

**Methods:**

Informed by a literature review, surveys, and consultation with men with a lived experience of suicidal thoughts and/or behaviours, we designed five video-based messages that will be used in this five-arm randomised controlled trial. A total of 380 (76 per arm) men aged 18 years or older with suicidal thoughts who are not currently accessing formal mental health services will be recruited online and randomly assigned to watch one of the five web-based video messages. After viewing the video, men will be presented with information about four existing Australian support services, along with links to these services. The primary outcome will be help-seeking, operationalised as a click on any one of the four support service links, immediately after viewing the video. Secondary outcomes include immediate self-reported help-seeking intentions in addition to self-reported use of the support services during a 1-week follow-up period. We will also use the Discrete Choice Experiment methodology to determine what aspects of support services (e.g. low cost, short appointment wait times) are most valued by this group of men.

**Discussion:**

This study is the first to evaluate the effectiveness of a brief web-based video messaging intervention for promoting engagement with existing support services among men with suicidal thoughts who are not currently receiving formal help. If found to be effective, this would represent a scalable, cost-effective approach to promote help-seeking for this at-risk population. Limitations and strengths of this study design are discussed.

**Supplementary Information:**

The online version contains supplementary material available at 10.1186/s13063-024-08308-1.

## Background

Suicide is a serious public health issue, and men are at disproportionately high risk [1]. In most Western countries including Australia, Canada, the UK, and the US, men are nearly three times more likely than women to die by suicide [2, 3]. Some of the disparity in suicide rates between men and women may be attributed to men being less likely to seek help for suicidal thoughts and behaviours. Research indicates that, in the year preceding suicide, 58% of women had contacted mental health services, whereas only 35% of men had done so [4]. The low service engagement among men with suicidal thoughts or behaviour has been suggested to be related to high self-reliance, the absence of a formal mental health diagnosis, lack of interpersonal support, and past negative experiences with formal health services [5, 6]. Given that suicide is an irreversible tragedy with profound impacts on individuals and their families, it is crucial to develop new approaches that more effectively target men who are experiencing suicidal thoughts and/or behaviour but are not actively seeking or receiving support services. Here, we refer to this at-risk population as those who are ‘under the radar’ of support services.

Most of the research on interventions aimed at promoting formal mental health help-seeking has to date, focused on using psychoeducation or cognitive behavioural strategies to improve mental health literacy [7, 8], reduce stigma related to help-seeking [9], or enhance motivation to engage with services [10]. Face-to-face delivery has been by far the most widely used approach to implementing these interventions, with interventions typically delivered by health professionals, case managers, or other community-based practitioners [11]. Although meta-analyses show some evidence of statistically significant effects of these interventions on help-seeking intentions and behaviour, most trials use an inactive or no control condition, and for the few that do use an active control comparator, non-significant effects are observed [11]. Importantly, these interventions have predominantly focused on promoting help-seeking among individuals with mental health problems who are already in contact with a service. This represents a significant implementation barrier for promoting help-seeking among men with suicidal thoughts/behaviour since many do not have a formal mental health diagnosis and are not engaged with formal services [12]. Furthermore, few prior studies have assessed the effectiveness of help-seeking interventions that are designed to address the specific barriers that prevent men from engaging with formal support services. Accordingly, new approaches that are tailored towards the unique needs of men who are ‘under the radar’ are needed.

The Internet offers a unique platform for delivering a wide range of digital assistance, which is particularly beneficial for individuals who may hesitate to seek help in person due to concerns about privacy or logistical obstacles. Digital help encompasses various forms, including but not limited to the provision of health information, connection with supportive communities of others with shared experiences, triage services, as well as automated or blended therapeutic interventions [13]. Notably, web-based video messages have been found to be highly engaging compared to other content formats such as online images or posts when it comes to delivering health information [14]. However, the application of video messages in the field of suicide prevention is an area that requires further exploration and investigation.

## Objectives

The primary aim of the Under the Radar trial is to determine which of five promotional messages is most effective at encouraging men with suicidal thoughts, who are ‘under the radar’, to engage with online support services. Five web-based video messages have been developed and will be evaluated in a five-arm randomised controlled trial. Three of the video messages were developed based on themes derived from reviews of the literature [5], a survey of help-seeking in men at risk of suicide [12], and through qualitative interviews with men experiencing suicidal thoughts [15]. Accordingly, these videos reflect the personal experiences and key issues of concern for men who do not access help for their suicidal thoughts. Two control video messages were also created, which provide either generic help-seeking advice or statistics on suicide. We hypothesise that video messages tailored to the concerns and personal experiences of men who are under the radar will be more effective at promoting engagement with support services than either generic help-seeking messaging or to factual data around suicide deaths and attempts.

A secondary aim of this study is to understand what attributes of support services men with suicidal thoughts value most. We will assess these service preferences using the Discrete Choice Experiment (DCE) methodology, which is popular in the field of health economics [16, 17]. DCEs involve presenting individuals with pairs of hypothetical support services that vary on a set of attributes (e.g. cost) and attribute levels (e.g. high cost vs. free). Participants are asked to select which service out of each pair they prefer. Based on participants’ pattern of choices, the relative preferences for the different attributes and levels can be estimated. This methodology will provide us with critical insights into the service attributes that are most appealing to men who are experiencing suicidal thoughts, as well as attributes that may hinder men’s willingness to engage with a service.

Together, the findings from this study are expected to yield valuable insights into how to encourage help-seeking and provide better support for men who are experiencing suicidal thoughts. Additionally, the findings from the trial may serve as the foundation for a nationwide communication campaign aimed at preventing suicide in men and strengthening partnerships with organisations dedicated to suicide prevention.

## Methods

### Trial design and setting

**Fig. 1:**
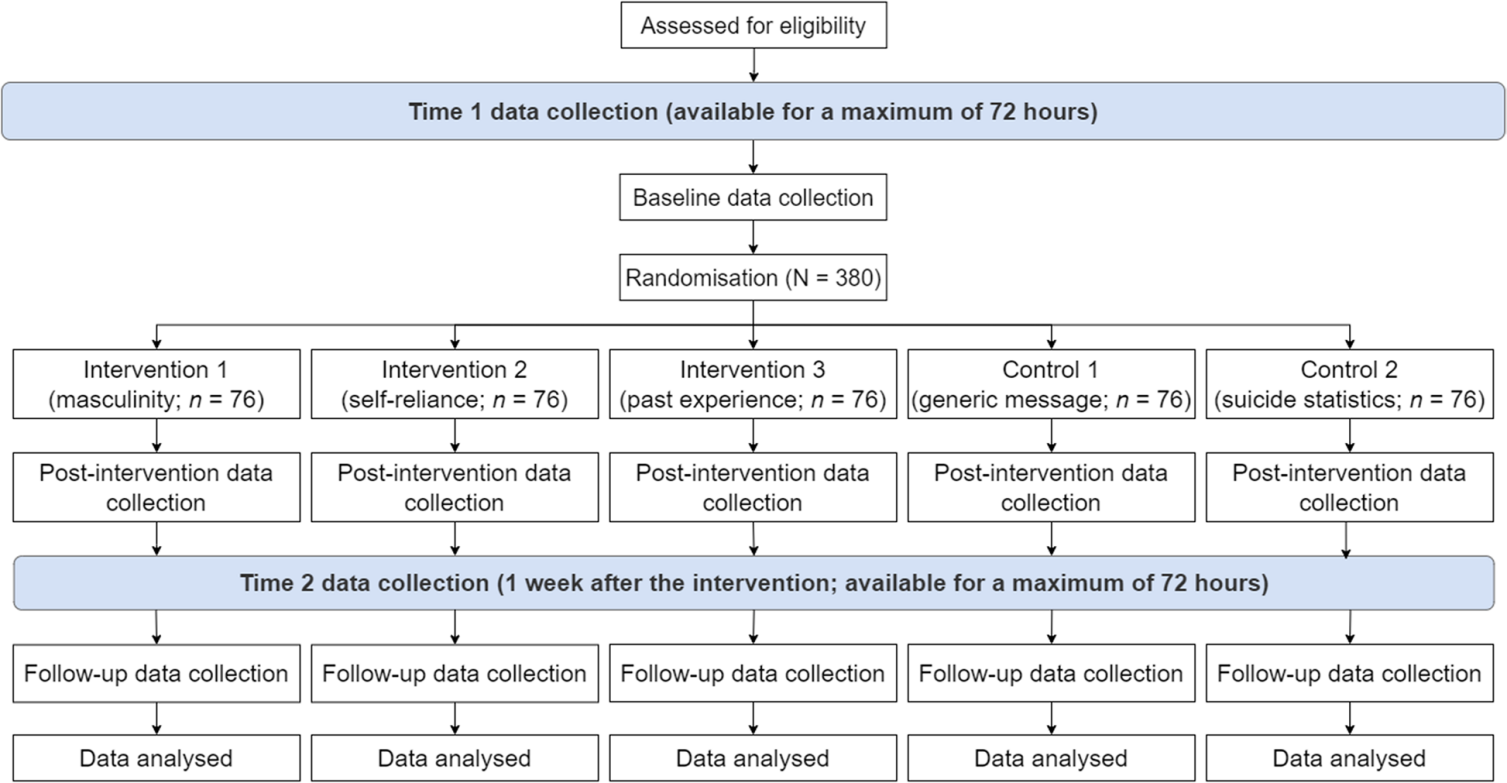
Trial flow

This is a five-arm randomised controlled superiority trial with three active arms and two control arms. Participant recruitment, administration of study procedures, and data collection will be conducted entirely online. Once enrolled in the study, participants will be randomly assigned to view one of five video message interventions; three of which test evidence-based messaging derived from themes in the prior literature, and two control videos that promote general help-seeking and suicide awareness. After viewing the videos, participants will be presented with a directory containing information about four mental health support services available in Australia, which have been selected for inclusion in this study based on their specialisation in providing mental health support for men, for their national reach, and for their range of service offerings (e.g. online help, telephone support, etc.). The four services are MensLine Australia, Mosh, Roses in the Ocean, and SANE Australia. Participants will be invited to click on a link to one or more of these services to find out more about what the service offers. There are three measurement occasions during the trial: baseline, post-intervention (immediately after viewing the video), and a 1-week follow-up (see Fig. 1).

### Patient and public involvement statement

A group of men with experience of suicidal thoughts, past history of trauma, and hesitancy against seeking help from formal mental health services participated in workshops and design exercises during the initial planning phase of this study. These men provided perspectives on the needs and views of those ‘under the radar’. Some of these men were also involved in quantitative and qualitative studies that were conducted prior to, and which informed, the design of the current study. All men were supported by the Black Dog Institute’s lived experience team and were paid for their participation.

### Sample and recruitment

A total of 380 men who are experiencing suicidal thoughts and who are not in receipt of formal mental health care will be recruited for this trial. Recruitment will be done using a social media drive involving advertisements posted on Facebook, Twitter, and Instagram. This recruitment strategy has proven successful in reaching our target population in prior survey and qualitative interview studies that were conducted during earlier phases of this project [12]. This initial contact will involve minimal real or perceived coercion or pressure to participate as the research team will not have direct contact with potential participants. Recruitment for the trial started on the 24th of July 2023. Participants will be reimbursed for their participation by means of two $20 GiftPay vouchers (total of $40)—one for completing the post-study survey and one for completing the follow-up survey. The reimbursement is being offered in acknowledgement of participants’ time and effort spent on completing the surveys and viewing the study intervention (video message). The gift vouchers will be provided to participants via the email address they provided after they were deemed eligible to participate in the study.

### Eligibility and screening

Participants will be eligible to participate if they (1) identify as a man, (2) are fluent in English, (3) are currently living in Australia, (4) have had thoughts of dying or hurting themselves in the past 6 months, and (5) have not accessed formal mental health services (including counsellors, general practitioners, psychologists, psychiatrists, and other mental health professionals) in the past 6 months.

Screening will be conducted online. First, participants who are interested in taking part in the study will click on a link in the recruitment advertisements. This link will take them to a study landing page where they will have the opportunity to read more about the study, view the participant information statement and consent form, and register to take part in the trial. After providing online consent, participants will answer a brief online screening survey to assess their eligibility. Those who are deemed eligible will continue on to the baseline questionnaire. Once participants enrol into the study, they are permitted to engage in any form of treatment they wish.

### Random allocation and blinding

Randomisation to the five trial arms will be conducted on a 1:1:1:1:1 ratio using a computerised algorithm. A blocked approach to randomisation will be implemented (block size of 10), using age as a stratifying variable (three stratums: age 18 to 35; age 36 to 64; age 65 or older). Participants will be randomised after consent has been provided and eligibility has been confirmed.

Participants will not be informed about the condition (intervention or control) to which they are assigned. The chance that participants will discern their condition assignment is low as the intervention and control video messages are presented in the same format. Operational staff involved in day-to-day participant management will be unblinded because the nature of the interventions requires that they cannot easily be concealed. All other investigators will be blinded to intervention assignment throughout the study period.

### Active conditions: videos portraying themes derived from studies on barriers to help-seeking for men with suicidal thoughts

The three key video messaging interventions will portray content as follows: (1) one video emphasises the positive elements of masculinity and conveys the message that seeking help requires strength, rather than constituting a weakness; (2) a second video challenges the idea that self-reliance is the best way to manage suicidal thoughts, and articulates how help-seeking facilitates problem-solving and development of practical solutions; and (3) a third video acknowledges that prior negative experiences with support services can act as a barrier to reaching out for help again, but emphasises the potential benefits to be gained by trialling services to find one that works.

The themes and content of the video messages were designed based on the quantitative and qualitative findings from earlier phases of the project [5, 12, 15, 18–21]. The video messages were of high-quality production and created by an award-winning filmmaker. Each video is approximately 2 min in length and is centred around a Caucasian, middle-aged male actor, who portrays first-hand experience of having suicidal thoughts. All videos are set in a domestic environment and depict the actor performing everyday tasks (e.g. making a cup of coffee, using tools to fix a skateboard) while speaking to the camera. Each video message ends with the actor encouraging the viewer to consider the support options provided. The actor’s age and ethnicity were chosen to match the demographic characteristics of our target population (in Australia, the largest number of men to die by suicide are Caucasian and aged 25–44 years).

### Control conditions: videos portraying generic messages around help-seeking and suicide awareness

The two control video messages portray the following content: (1) one video uses messaging similar to that used on the websites of existing suicide prevention organisations, and promotes help-seeking and general awareness of the availability of support services for individuals experiencing suicidal thoughts; (2) a second video aims to increase the viewers’ general knowledge of the prevalence of suicide in Australia by presenting Australian suicide statistics in the style of a news presenter. These two control video messages are of the same length as the active intervention video messages and are presented in the same style and with the same actor.

### Assessment schedule

**Fig. 2:**
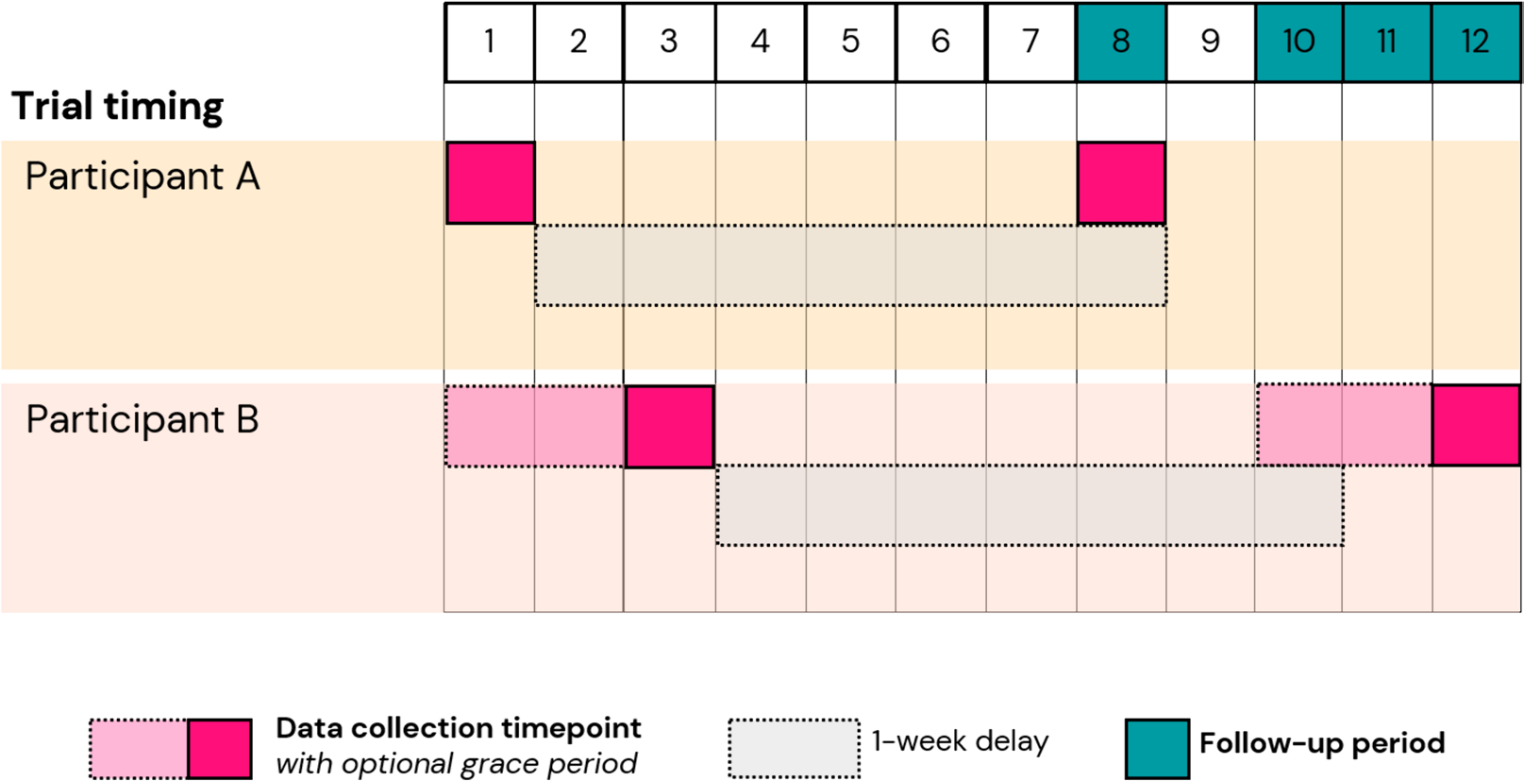
Trial timing

Outcomes will be collected at three time points: baseline (day 1), post-intervention (day 1), and 1-week follow-up (day 8). Data collection will be conducted entirely online through an automated trial platform. Immediately following completion of the screening survey, eligible participants will complete the baseline questionnaires, view the video messaging intervention, and complete the post-intervention questionnaires. One week later, participants will receive an SMS prompt inviting them to complete the 1-week follow-up questionnaires. Participants have a maximum of 72 h to complete baseline and post-intervention, and another 72 h to complete follow-up. The DCE survey is available for the duration of the trial. See Fig. 2 for the trial timing where participant A completes all tasks at the first opportunity, while participant B uses all of the grace period before completing the tasks.

During the baseline assessment, only a small number of questionnaires will be administered. This approach aims to minimise the impact of pre-exposure to information on the type of support that is sought. A minimal baseline questionnaire battery also more closely replicates the conditions of mass media campaigns, where individuals approach the content with limited prior knowledge of what the campaign is about.

### Outcome measures

The full schedule of assessments is shown in Table 1.

**Table 1:** Summary of the primary, secondary, other outcome measures, and data collection time points

Outcome measures	Assessment	Baseline	Post-intervention	One-week follow-up
*Primary outcome*				
Help-seeking behaviour	Click-throughs	–	✓	–
*Secondary outcomes*				
Use of support services	Self-report item	–	–	✓
Help-seeking intentions	Self-report item	–	–	✓
Service preferences	Discrete Choice Experiment	–	✓	–
*Exploratory outcomes*				
Awareness of services	Self-report item	–	✓	–
Frequency of services chosen	Click-throughs	–	✓	–
Intervention acceptability	Theoretical Framework of Acceptability Questionnaire	–	✓	–
Time spent viewing video	Seconds of video play	–	✓^a^	–
Suicidal distress	University of Washington Risk Assessment Protocol	✓	✓	–
Demographics	Self-report item	✓	–	–
Suicidal thoughts	Suicidal Ideation Attributes Scale	–	✓	–
General mental wellbeing	Short Warwick Edinburgh Mental Wellbeing Scale	–	✓	–
Psychological distress	Kessler Psychological Distress Scale	–	✓	–
Loneliness	Three-Item Loneliness Scale	–	✓	–
Anhedonia	Snaith-Hamilton Pleasure Scale	–	✓	–
Physical and mental health	Self-report item	–	✓	–
Barriers to services	Barriers to Access to Care Evaluation Scale	–	–	✓
*Process outcomes*				
Service engagement patterns	Service usage data provided by four support services	–	–	✓

^a^Measured during intervention delivery

#### Primary outcome

##### Help-seeking behaviour

The primary outcome is help-seeking behaviour, operationalised as a click on the link to at least one of the four support services presented to participants (i.e. MensLine Australia, Mosh, Roses in the Ocean, SANE Australia) immediately after the video is shown.

#### Secondary outcomes

##### Use of support services

Use of the four support services will be assessed at the 1-week follow-up using a single item that asks participants whether they have used any of the four support services in the past week (response options: ‘Yes’ or ‘No’).

##### Help-seeking intentions

Help-seeking intentions will be measured at the 1-week follow-up assessment using an item that asks participants to rate how likely they would be to access help from one of the four support services if they experienced suicidal thoughts in the future. Participants will respond using a 7-point Likert scale ranging from 1 (extremely unlikely) to 7 (extremely likely). Four separate questions will be used to assess this outcome, with a separate question referring to each of the four support services shown. The highest score of the four questions will be used to indicate participants’ intentions to seek help from one of the four support services in the future.

##### Service preferences

Preferences for suicide prevention services will be elicited through a DCE [16, 17]. DCEs are a survey-based method used to understand how people make choices among different service options with varying attributes. DCEs create hypothetical choice situations, where each choice (service or product) is described by a set of attributes (characteristics) and levels. The DCE will allow inferences to be drawn about the relative importance of suicide prevention service attributes by observing the trade-offs that respondents make when choosing their preferred service.

**Fig. 3:**
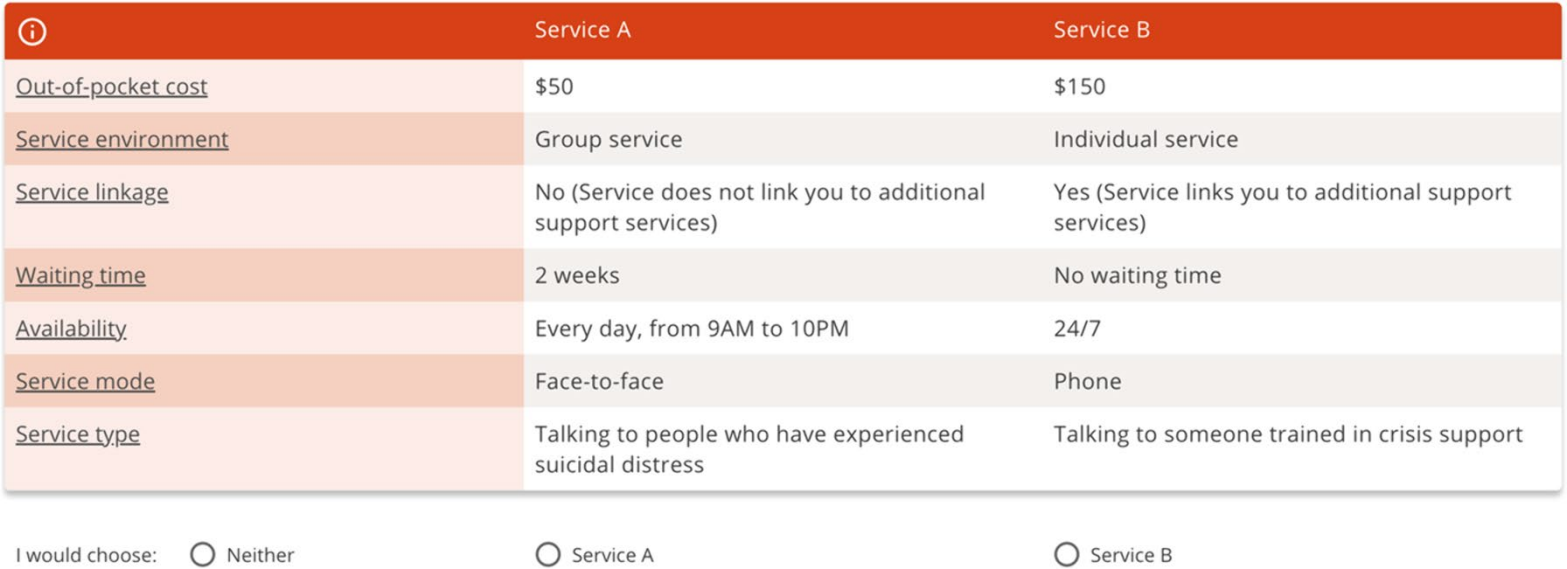
Example item from the Discrete Choice Experiment

A list of seven service attributes, each with varying levels, has been constructed based on a literature review and refined through interviews with men with lived experience of suicidal thoughts (*n* = 7). The final list of attributes is out-of-pocket cost, service type (i.e. self-help resources, peer support, counselling, healthcare), service mode (i.e. online, phone, face-to-face), waiting time, availability, service environment (i.e. individual or group), and service linkage (i.e. linking to services helping with employment, finances, housing, relationships). Attribute levels will be altered across the scenarios presented and respondents will be asked to choose their preferred hypothetical suicide prevention service. Each scenario will include two suicide prevention services constructed using the seven attributes and an additional option to choose neither. Scenarios will be presented to participants in the format shown in Fig. 3.

#### Exploratory outcomes

A range of exploratory measures will be used to assess aspects of service awareness and use, as well as key demographic and clinical moderating variables.

##### Prior awareness and use of the four support services

Participants’ awareness and use of the four support services prior to the study will be assessed using two closed-choice questions presented at the post-intervention assessment: (1) ‘Have you heard of any of the following services (MensLine Australia, Mosh, Roses in the Ocean and SANE Australia) before taking part in this study?’ and (2) ‘Have you used any of the following services (MensLine Australia, Mosh, Roses in the Ocean and SANE Australia) before taking part in this study?’ Response options: ‘Yes’ or ‘No’.

##### Support service chosen most frequently

We will evaluate which of the four support services is chosen most frequently by calculating the number of times the link to each support service is clicked at post-intervention.

##### Acceptability of the video messaging intervention

The Theoretical Framework of Acceptability questionnaire (TFA) [22] will be used to evaluate participants’ perceptions of the acceptability of the video messaging intervention at the post-intervention assessment. The TFA contains eight items to evaluate the acceptability of healthcare interventions according to their different features (e.g. the perceived effectiveness of the intervention, effort required to engage with the intervention, etc.). Participants rate the extent to which they agree or disagree with each item on a 5-point Likert scale. The distribution of scores will be examined and, based on this distribution, either a mean or a median score will be computed. Higher scores indicate greater acceptability of the intervention. Other aspects of acceptability will also be examined at post-intervention using open-ended qualitative questions: ‘What about the video message appealed to you?’ and ‘What about the video message did not appeal to you?’.

##### Time spent viewing the video

Participants can start, stop, and skip the video message at any time. We will capture the total number of seconds of the video that participants viewed.

##### Impact of intervention on participants’ stress

A single stress item from the University of Washington Risk Assessment Protocol (UWRAP) [23] will be used to evaluate the possible adverse effects of the intervention. The UWRAP is a widely used tool for assessing and managing suicide risk. The stress item in the UWRAP is designed to evaluate the level of stress a person is experiencing. The scale ranges from 0 (no stress) to 7 (highest level of stress). The rating is based on an individual’s subjective experience of stress.

##### Demographics

Demographic variables, including age, current relationship status, highest level of education completed, employment status, and geographic location (i.e. postcode), will be collected at baseline.

##### Severity of suicidal ideation and past attempts

The Suicidal Ideation Attributes Scale (SIDAS) [24] will be used to measure the severity of suicidal thoughts in the past month. This will be assessed at the post-intervention assessment. The SIDAS consists of five items that assess the frequency of suicidal thoughts, the controllability of suicidal thoughts, the closeness an individual has come to making a suicide attempt, the level of distress associated with the thoughts, and the impact of these thoughts on daily functioning. Each item is rated on an 11-point scale (0–10). Item two (controllability) is reverse scored. Total scores on the SIDAS range from 0 to 50, with higher scores indicating more severe suicidal ideation. Previous suicide attempts and self-injury will also be assessed using two questions that ask participants to indicate whether they have attempted suicide (a) in their lifetime and (b) in the past 30 days. Participants respond on a 3-point Likert scale: 0 (‘No, never’), 1 (‘Yes, once’), and 2 (‘Yes, more than once’). Participants are also asked to report the date of their most recent attempt.

##### Wellbeing

The Short Warwick Edinburgh Mental Wellbeing Scale (SWEMWBS) [25] is a 14-item self-report scale that will be used to assess mental well-being at the post-intervention assessment. The SWEMWBS asks participants to rate how often they have experienced different thoughts and emotions over the past 2 weeks on a 5-point Likert scale ranging from 1 (‘None of the time’) to 5 (‘All of the time’). Raw item scores are summed and converted to a metric total score using the SWEMWBS conversion table. Total scores range from 7 to 35, with higher scores indicating better mental well-being.

##### Psychological distress

The Kessler Psychological Distress Scale (KTEN) [26] is a 10-item self-report scale that will be used to assess levels of psychological distress at the post-intervention assessment. It consists of ten questions that ask participants about their feelings and experiences over the previous 4 weeks on a 5-point Likert scale, ranging from 1 (‘None of the time’) to 5 (‘All of the time’). The raw item scores are summed, resulting in a total score ranging from 10 to 50. Higher scores indicate higher levels of psychological distress.

##### Loneliness

The Three-Item Loneliness Scale (TILS) [27] will be used to measure self-reported levels of loneliness at the post-intervention assessment. Items ask: ‘How often do you feel that you lack companionship?’ (Relational connectedness); ‘How often do you feel left out?’ (Collective connectedness); and ‘How often do you feel isolated from others?’ (General isolation). Response categories are as follows: 1 (‘Hardly ever’), 2 (‘Some of the time’), and 3 (‘Often’). The raw item scores are summed to compute a total score, with higher scores indicating greater loneliness.

##### Anhedonia

The Snaith-Hamilton Pleasure Scale (SHAPS) [28] will be used to assess anhedonia. It contains 14 items, covering four domains of hedonic experience: interest/pastimes, social interaction, sensory experience, and food/drink. Each item asks participants to rate how much they agree or disagree with statements of hedonic response in pleasurable situations (for example, ‘I would enjoy my favourite television or radio program’) on the basis of their experience in the ‘last few days’. Items are rated on a 4-point Likert scale, ranging from 1 (‘Strongly disagree’) to 4 (‘Strongly agree’). The raw item scores are summed to compute a total score. Scores range from 14 to 56, with higher scores indicating more severe levels of anhedonia [29].

##### Physical and mental health status

Two questions will be administered at the post-intervention assessment to assess physical and mental health status. These questions will ask participants whether they have previously been diagnosed with a chronic physical health condition or a mental health condition by a professional (e.g. a doctor). If there is an existing mental health condition, further questions on the diagnosis and duration of the condition will be asked.

##### Barriers to accessing healthcare services

The Barriers to Access to Care Evaluation Scale (BACE) [30] will be used to assess the severity of the barriers that prevent individuals from accessing healthcare services. This will be measured at the 1-week follow-up assessment. This scale consists of 30 items, categorised into subscales of stigma-related and non-stigma-related barriers associated with the utilisation of mental health services. Each item is rated on a 4-point Likert scale, ranging from 0 (‘Not at all’) to 3 (‘A lot’). The results are reported as the percentage of endorsement for each barrier, with higher scores indicating greater prevalence and impact of these barriers.

#### Process outcomes

##### Patterns of service engagement

We will examine markers of service engagement among men who access one of the four support services. These markers will be derived using data provided directly by the service. Although the nature and extent of data provided by each service will differ slightly, the types of data provided will broadly cover aspects of service use (e.g. number of sessions attended with a psychologist), engagement (i.e. whether an individual completed a course of treatment), and appeal (e.g. consumer-rated usefulness of the service). This data will be provided at an aggregated level across all trial arms. Approaches to analysing this data will depend on the sophistication of data capture across the four services.

### Sample size and power

Based on previous empirical studies of mental health service usage, we estimated that 28% of the participants randomised to one of the three target intervention video messages would click on the link of at least one of the four support services (primary outcome). By contrast, we estimated only 8% of participants randomised to one of the two control video messages would click on the link to at least one support service. Accordingly, we calculated that *n* = 76 participants per trial arm (*N* = 380 total) would provide 80% power to detect our effect of interest with a type I error rate of *α* = 0.05.

### Statistical analysis

#### Analysis of primary outcome

The primary outcome will be analysed using binary logistic regression. These analyses will be conducted using R (version 3.6.3) in R Studio (version 1.2.5033). Models will evaluate the effects of the specific video messaging interventions on help-seeking at post-intervention (i.e. whether the participant clicked on at least one of the four support service links). We will build two models to evaluate specific intervention contrasts:

1. Model 1 will assess differences between the three target video messaging conditions and the two control video messaging conditions by using a single *Intervention* term coded as 1 for the three target interventions and 0 for the two control interventions.
2. Model 2 will assess differences between the three target video messaging conditions by entering two terms: *Video 1* (coded 1 for target video 1 and coded 0 for the other two target videos) and *Video 2* (coded 1 for target video 2 and coded 0 for the other two target videos).

Pending the outcomes of these models, we will perform exploratory analyses to examine differences between specific intervention conditions. As the outcome will be measured at a single time point, no imputation of missing data will be performed. The study sample considered for inclusion will be defined as all participants who meet eligibility criteria and who were randomised to one of the five intervention conditions.

#### Analysis of secondary outcomes

Use of the four services within the 1-week follow-up period will be analysed using the same approach as used for the primary outcome. Help-seeking intentions, which are measured on a 7-point Likert scale, will be measured using ordinal logistic regression. In the case that some levels on this 7-point scale are underrepresented, we will consider combining adjacent categories as appropriate.

We will analyse respondents’ choices made within the DCE by estimating the conditional logit models [31] and mixed logit models [32]. These analyses will be performed using STATA (version 18). These models can be interpreted via the Random Utility Theory which assumes that individuals will choose the alternative that provides them with the highest utility. We will also estimate the Willingness to Pay (WTP), which is the dollar amount individuals are willing to pay for service changes. Relative importance of attributes will also be assessed to understand the relative impact that an attribute has on choice. For example, the preference analysis results will allow us to explore whether men prefer one service type (e.g. peer support) over another (e.g. seeing a psychiatrist). The WTP results will indicate the dollar amount men are willing to pay to receive one service compared to another (e.g. they might be willing to pay $50 more to receive services individually rather than in a group setting). The relative importance results will allow us to understand the ranking of the suicide prevention attributes (e.g. the most important factor affecting choice may be service type, followed by waiting time, cost, etc.). To explore observed heterogeneity, sub-group analyses will be undertaken by interacting the preference weights with a range of socio-demographic characteristics including age, educational attainment, employment status, income levels, etc. This will allow us to understand why individuals in one sub-group (e.g. higher educational attainment) may have different preferences compared to those in another sub-group (e.g. lower educational attainment).

#### Exploratory analyses

We will also perform exploratory analyses to assess the potential moderating effects of demographic and clinical variables collected, such as participant age and the severity of their suicidal ideation, as well as the length of time participants spent viewing the video message. These analyses will be performed by entering interaction terms into the abovementioned models. Moderators will be assessed one at a time and using separate models.

### Risk management

To minimise the risk of any potential discomforts caused by participating in the current study, participants will be provided with information about the study prior to participating. Specifically, in the participant information statement and consent form, they will be provided with an overview of the study aims, will be informed that they will be randomly assigned to view one of five brief videos about suicide prevention (although not informed in detail about the specific messaging used in the videos), and the types of questions included in the study surveys, including those that have the potential to cause distress. On the recruitment landing page and every page of the trial website, participants will be provided with phone numbers for Lifeline and the Suicide Call Back Service, to ensure that participants have 24/7 access to support if they wish to access it.

Regardless of the condition to which participants are assigned, links to support services participating in the current study as partner organisations (MensLine Australia, Mosh, Roses in the Ocean, and SANE Australia) will be provided immediately after the participants are exposed to the video message. These organisations have agreed to provide services to participants referred to them during the study. If participants do not wish to answer a particular question, they will be able to either skip the question and go to the next question (for questions that are optional), or they can withdraw from the study by closing the web browser containing the survey (for questions that are mandatory). Participants will also be able to take a break at any time and come back to answering questions when they feel ready to do so within the given study period. Given that there is a known stigma associated with suicide or self-harming behaviour, there is a risk that participants may be subject to, or perceive, discrimination, stigma, or other social harm. By asking participants to create a secure and confidential account for this study using an active email address, these risks will be minimised.

In addition to the help-seeking resources provided following the video message, at the end of the intervention and the surveys, participants will be provided with options to contact other crisis support services (i.e. Lifeline). An agreement is also in place for a ‘clinician on duty’ (i.e. a clinical psychologist affiliated with the research team) to provide brief support to any potential participants experiencing a high level of emotional distress. To implement this, the research team will maintain a psychological safety response register where any disclosures of risk are recorded. If a participant discloses risk, the research team will notify the clinician on duty, who will then attempt to contact the participant via phone or email within 1 business day to offer a 30-min follow-up phone call to assess risk and offer support options.

All protocol deviations will be reported to the Principal Investigator and logged in a protocol deviations spreadsheet. All protocol modifications will be submitted to the UNSW Human Research Ethics Committee (HREC) for review and approval prior to implementation. For any events identified as serious and/or unexpected, the Principal Investigator will provide written notification of the event to the sponsor’s Research Ethics Compliance Support Office within 24 h. The UNSW HREC also monitor trial conduct via an annual report that is completed by the Principal Investigator.

### Privacy, confidentiality, and data management

Sensitive data will be collected in this study, including participants’ responses to the survey, clicks on links to the four support service partners, as well as self-reported use of these support services. To ensure the privacy of this data, each participant will be automatically assigned a unique study identification number at the time of registration on the research platform. In addition, a unique code will be delivered to the registered email address during each log-in attempt. When online survey data are exported for analysis, the research team will remove the identifiable information from the initial data set. A deidentified extract of the data will be downloaded for analysis on a shared drive, which will be password protected, encrypted, and approved by the University of New South Wales for storing highly sensitive data. The file used for analysis will only include the unique study ID and raw research data. Only named study personnel will have access to any identifiable information.

### Data monitoring

A Trial Operations Group comprised of Project Managers, Research Officers, Software Engineers, and a Data Manager will meet weekly. Its members will be responsible for data verification, responding to any participant queries/concerns, and any technical issues with the trial platform. Members of the Trial Operations Group will also be responsible for monitoring disclosures of risk or heightened distress from participants and initiating appropriate safety procedures.

An Internal Management Committee (IMC) comprised of staff from the coordinating centre, including the Principal Investigator, Project Leads, Project Manager, Postdoctoral Researchers, Research Officers, and the Data Manager, will meet fortnightly. The purpose of the IMC will be for the Lead Investigator and Project Leads to provide oversight of the trial, and for staff involved in managing trial operations to provide regular updates, and seek guidance on any unresolved issues from the Trial Operations meetings. Stakeholders and Lived Experience Advisors who were consulted during the planning stage of the trial will be updated on trial progress via email.

A Data Safety Monitoring Board (DSMB) has been established to assess the safety and efficacy of the trial and provide recommendations about whether to continue, modify, or stop the trial. The DSMB consists of a chairperson and two members (one of whom is the trial biostatistician) who are independent of the trial sponsor. The DSMB will meet after the recruitment of the first 100 study participants and at study closeout. Aggregated data will be presented to the DSMB, including safety parameters (SIDAS, UWRAP, number of distressed participants contacting via the study email account), compliance-related data (analytics data related to clicks on the links to the services and incomplete/complete views of the video messages), and if applicable, any adverse events reported in the trial safety monitoring register.

### Dissemination policy

The research results will be reported in academic journals. Results will also be presented at relevant academic conferences. All authors must fulfil the International Committee of Medical Journal Editors (ICMJE) requirements of authorship. Deidentified findings will be disseminated to partner organisations, other researchers, and participants through broader channels such as newsletters, websites, and social media. Participant confidentiality will be maintained by only reporting aggregate results. In all reports, participants will not be individually identifiable.

## Discussion

The Internet offers an excellent platform for disseminating health information; it can provide support to a large population in a cost-effective and timely manner, while maintaining high fidelity [33]. In this study, we introduce a novel approach to engage ‘under the radar’ men with suicidal thoughts and/or behaviour who are not currently accessing services. We employ video messages based on personal stories shared by men in our prior work who have lived through suicidal experiences. This intervention holds significant potential to promote health service engagement among suicidal men, capitalising on the advantages of online video messages. These messages provide a non-judgmental and confidential channel of communication, allowing men to share their knowledge on overcoming barriers to seeking help.

The video messages utilised in this study were developed in close collaboration with men who have lived experience with suicidal thoughts and/or behaviour and were created by a videographer with previous experience in producing work that explored mental health issues in the community. Through a combination of qualitative and quantitative research methods conducted during the early phases of the Under the Radar project, we sought to understand and address their primary concerns. These video messages were specifically designed to target these concerns. Findings from the trial are likely to provide preliminary understandings of whether certain messages outperform others in promoting service engagement among men experiencing suicidal thoughts and/or behaviour. Moreover, the trial evaluates health service engagement outcomes at both behavioural and intentional levels, providing a comprehensive understanding of how the intervention may influence service usage engagement. The findings from this trial have the potential to offer valuable insights into the differential impact of health interventions on behaviour and intentions in the field of health behaviour promotion.

If the video messages prove effective in increasing service engagement among men with suicidal thoughts and/or behaviour, they can be utilised in mass media campaigns dedicated to suicide prevention in men. Successful implementation of video messaging interventions would serve as a valuable addition to existing interventions for suicide prevention in men, particularly for those who may have reservations about seeking help through conventional channels.

## Trial status

Recruitment began on the 24th of July 2023. The trial will run until 380 Under the Radar men are recruited. Protocol version 2 dated 12/03/2024.

### Supplementary Information

Additional file 1. Participant information statement and consent form.

Additional file 2. SPIRIT checklist for Trials.

## Data Availability

The datasets and intervention materials are available upon request to the corresponding author after completion of the trial. Aggregated data will be communicated by publications on peer-reviewed journals and poster or oral presentations at conferences.

## Abbreviations

BACE: Barriers to Access to Care Evaluation Scale
DCE: Discrete Choice Experiment
KTEN: Kessler Psychological Distress Scale
SHAPS: Snaith-Hamilton Pleasure Scale
SIDAS: Suicidal Ideation Attributes Scale
SWEMWBS: Short Warwick Edinburgh Mental Wellbeing Scale
TFA: Theoretical Framework of Acceptability
TILS: Three-Item Loneliness Scale
UK: United Kingdom
US: United States
UWRAP: University of Washington Risk Assessment Protocol
WTP: Willingness to Pay
