# Supplementary material for: A web-based video messaging intervention for suicide prevention in men: study protocol for a five-armed randomised controlled trial
Source: Trials. 2024 Jul 9;25:466. doi: 10.1186/s13063-024-08308-1 (PMC11234748; doi:10.1186/s13063-024-08308-1)
Supplement: Supplementary file 1 — Additional file 1. Participant information statement and consent form. [file 13063_2024_8308_MOESM1_ESM.pdf]

PARTICIPANT INFORMATION STATEMENT AND CONSENT FORM

A randomised controlled trial of web-based video messaging interventions for suicide prevention in males (Under the Radar)

Professor Helen Christensen

1. What is the research study about?

The Black Dog Institute is inviting men who have thought of dying or hurting themselves in the past six months to participate in a trial to help us understand how to better support men in suicidal distress. The trial aims to assess if video-based messages tailored to men's characteristics and situations will promote suicide prevention.

2. Who is conducting this research?

The study is being carried out by the following researchers:

- Professor Helen Christensen, Black Dog Institute/University of New South Wales
- Professor Katherine Boydell, Black Dog Institute/University of New South Wales
- Associate Professor Fiona Shand, Black Dog Institute/University of New South Wales
- Dr Jin Han, Black Dog Institute/University of New South Wales
- Professor Philip Batterham, Centre for Mental Health Research, Australian National University
- Professor Henry Cutler, Macquarie University Centre for the Health Economy
- Professor Svetha Venkatesh, Applied Artificial Intelligence Institute, Deakin University
- Dr Leonard Hoon, Applied Artificial Intelligence Institute, Deakin University
- Dr Aimy Slade, Black Dog Institute
- Ms Noura Saba, Macquarie University Centre for the Health Economy
- Dr Wu Yi Zheng, Black Dog Institute
- Ms Hiroko Fujimoto, Black Dog Institute
- Dr Alexis Whitton, Black Dog Institute/University of New South Wales
- Associate Professor Yuanyuan Gu, Macquarie University Centre for the Health Economy
- Dr Anam Bilgrami, Macquarie University Centre for the Health Economy

Research Funder: This research is being funded by the Medical Research Future Fund.

3. Inclusion/Exclusion Criteria

Before you decide to participate in this study, we need to ensure that it is ok for you to take part. The research study is looking to recruit people who meet the following criteria:

- 18 years of age or older
- Identify their gender as male
- Fluent in English
- Currently living in Australia
- Thought of dying or hurting themselves in the past six months
- No contact with formal mental health services (including General Practitioners, Psychologists, Psychiatrists and other mental health professionals) for mental health support in the past six months.

4. Do I have to take part in this research study?

Participation in this research study is voluntary. If you do not want to take part, you do not have to. If you decide to take part and later change your mind, you are free to withdraw from the study at any stage.

5. What does participation in this research require, and are there any risks involved?

If you agree to participate you will be asked to complete the following research procedures:

- Provide consent electronically by ticking the 'I consent' box at the bottom of the webpage

- Fill in a short survey (two minutes) to check your eligibility for this study.
- If you are eligible for the study, you will then be asked to provide your email address and first name, which will only be used for contacting you regarding the study. After validating your email address, you will be able to start your participation in the study. If you are not eligible to take part in the study, you will be informed and provided with information of support services.
- You will be directed to answer a few questions about yourself. Questions include ethnicity, language, living status (urban or rural, length of time living in Australia), education, employment, and distress levels. The survey takes about three minutes to complete.
- After completing these questions, you will be randomly allocated to watch one of five videos on suicide prevention: one of three tailored messages, a general message, or a control message. Each video lasts approximately 2 minutes. Please watch the video as soon as possible as the video will only be available for three days. After watching the video, you will be asked to answer a few questions related to suicide prevention, taking about two minutes to complete.
- You will then be invited to complete a survey, including questions on your experience in watching the video, physical and mental health, suicidal thoughts and behaviour, and preference for different suicide prevention services. The survey takes about 30 minutes to complete. After completing this survey, you will receive a $20 GiftPay voucher via email which can be used at a range of retailers.
- After one-week, you will receive an email invitation to a survey, asking about your health behaviour and perceptions towards suicide prevention in the past week. The survey takes about eight minutes to complete.
- After completing the follow-up survey, you will receive a second $20 GiftPay voucher via email.
- If we collect enough participants of similar profiles, there is a possibility that you might not be able to participate in the current study.

If you experience discomfort or feelings of distress while participating in the research, you can stop participating at any time. If you require immediate support or would prefer to receive support from someone not involved in the research, please contact your local GP, Beyond Blue (1300 22 4636), the Suicide Call Back Service (1300 659 467) or Lifeline (13 11 14).

6. What are the possible benefits of taking part?

We cannot guarantee or promise that you will receive benefits from participating in this research; however, possible benefits may include new knowledge on the available help-seeking resources. In addition, your participation will help us understand how to support other men with suicidal thoughts or behaviour in the community.

7. What will happen to information about me?

By signing the consent form, you consent to the research team collecting and using information about you for the research study. If you do not meet the inclusion criteria, no personal data will be stored on the servers. The research team will store the data collected from you for this research project for a minimum of 7 years after project completion. We will store information about you in a non-identifiable format on a UNSW Sydney approved IT platform and data is protected by encryption. The identifiable information (i.e. first name and email address) collected in this study will be used to contact you for consented activities only (i.e., surveys, e-gift card, research report). Data will be stored in re-identifiable format where any identifiers such as your name, address, date of birth will be replaced with a unique code. Identifiable information will be stored separately from your survey responses on the UNSW OneDrive, protected by password, accessed by named study personnel only.

The information you provide is personal information for the purposes of the Privacy and Personal Information Protection Act 1998 (NSW). You have the right of access to personal information held about you by the University, the right to request correction and amendment of it, and the right to make a complaint about a breach of the Information Protection Principles as contained in the PPIP Act. Further information on how the University protects personal information is available in the UNSW Privacy Management Plan.

8. How and when will I find out what the results of the research study are?
The research team intend to publish the results of the research study. All information published will be done in a way that will not identify you. If you would like to receive a copy of the results, you can let the research team know by adding your email within the consent form. We will only use these details to send you the results of the research.

9. What if I want to withdraw from the research study?
If you do consent to participate, you may withdraw at any time. You can do so by completing the 'Withdrawal of Consent Form' which is provided at the end of this document. Alternatively, you can call or email the research team and tell them you no longer want to participate. Your decision not to participate or to withdraw from the study will not affect your relationship with UNSW Sydney, the Black Dog Institute, Deakin University, and Macquarie University. If you decide to leave the research study, the researchers will not collect additional information from you. You can request that any identifiable information about you be withdrawn from the research project.

10. What if I have a complaint or any concerns about the research study?
If you have a complaint regarding any aspect of the study or the way it is being conducted, please contact the UNSW Human Ethics Coordinator:

Complaints Contact

**Position**	UNSW Human Research Ethics Coordinator
**Telephone**	+ 61 2 9385 6222
**Email**	humanethics@unsw.edu.au
**HC Reference Number**	HC220714

11. What should I do if I have further questions about my involvement in the research study?
The person you may need to contact will depend on the nature of your query. If you require further information regarding this study or if you have any problems which may be related to your involvement in the study, you can contact the following member/s of the research team:

Research Team Contact Details

**Name**	Dr Jin Han
**Position**	Research Fellow
**Telephone**	02 9065 9109
**Email**	undertheradar@blackdog.org.au

Chief Investigator

**Name**	Helen Christensen

**Position**	Professor
**Telephone**	02 9382 4530
**Email**	undertheradar@blackdog.org.au

Support Services Contact Details
If at any stage during the study, you become distressed or require additional support from someone not involved in the research please call:

**Organisation**	Lifeline<br>Crisis support and suicide prevention
**Telephone**	13 11 14 available 24/7
**Online chat**	www.lifeline.org.au/crisis-chat available 19:00 – 23:59 AEDT/AEST
**Web**	www.lifeline.org.au/get-help

**Organisation**	Suicide Call Back Service<br>Counselling and support for people affected by suicide
**Telephone**	1300 659 467 available 24/7
**Online chat**	www.suicidecallbackservice.org.au/phone-and-online-counselling/suicide-call-back-service-online-counselling available 24/7
**Web**	www.suicidecallbackservice.org.au

**Organisation**	Beyond Blue<br>Suport and information for people experiencing depression or anxiety
**Telephone**	1300 22 4636 available 24/7
**Online chat**	online.beyondblue.org.au available 11:00 – 23:59 AEDT/AEST
**Web**	www.beyondblue.org.au

PARTICIPANT INFORMATION STATEMENT AND CONSENT FORM

A randomised controlled trial of web-based video messaging interventions for suicide prevention in males (Under the Radar)
Professor Helen Christensen

Consent Form

You can click and download Participant Information Sheet and Consent Form for your reference.

By checking the options below:

- ☐ I understand I am being asked to provide consent to participate in this research study;
- ☐ I have read the Participant Information Sheet and confirm that it is written in a way I understand;
- ☐ I freely agree to participate in this research study as described and understand that I am free to withdraw at any time during the study and withdrawal will not affect my relationship with any of the named organisations and/or research team members;
- ☐ I understand that if necessary, I can ask questions and the research team will respond to my questions;
- ☐ I would like to receive feedback from the Institute on the results of this research (optional); Email address: ________________________________
- ☐ I provide my consent for my name and contact details to be retained in a register so I can be contacted about other research projects in the future (optional).
- ☐ I provide my consent for de-identified/aggregated data to be shared with support organisations who are partners on this research study (optional)

Note: If you change your email address, please email the research team: undertheradar@blackdog.org.au

Form for Withdrawal of Participation

I wish to **WITHDRAW** my consent to participate in this research study described above and understand that such withdrawal **WILL NOT** affect my relationship with UNSW Sydney, the Black Dog Institute, Macquarie University, and Deakin University.

- ☐ I am withdrawing my consent and I would like any identifiable information collected about me which I have provided for the purpose of this research study withdrawn.

- ☐ I am withdrawing my consent to participate in further components of this research and provide my permission for the research team to retain and/or use information collected about me which I have provided for the purpose of this research.

- ☐ I am withdrawing my consent and I understand that any information already published and/or not linked to my identity cannot be withdrawn from the research.

Participant Signature

Name of Participant (please print)	
Signature of Research Participant	
Date	

The section for Withdrawal of Participation should be forwarded to:

CI Name:	Professor Helen Christensen
Email:	undertheradar@blackdog.org.au
Phone:	02 9382 4530

**PARTICIPANT INFORMATION STATEMENT AND CONSENT FORM**	

PARTICIPANT INFORMATION STATEMENT AND CONSENT FORM

A randomised controlled trial of web-based video messaging interventions for suicide prevention in males (Under the Radar)

Professor Helen Christensen

Postal Address:	The Black Dog Mental Health Institute, Hospital Road, Randwick, NSW 2031
